# Recent Advances in Metal‐Based Catalysts for Nitrile Hydration to Amides: Mechanistic Aspects

**DOI:** 10.1002/tcr.202500155

**Published:** 2025-11-03

**Authors:** Fatima‐Ezzahraa Essebbar, Hicham Ben El Ayouchia, Hafid Anane, Salah‐Eddine Stiriba

**Affiliations:** ^1^ Laboratoire de Chimie Analytique et Moléculaire, LCAM Faculté Polydisciplinaire de Safi Université Cadi Ayyad Safi Morocco; ^2^ Instituto de Ciencia Molecular/ICMol Universidad de Valencia Valencia Spain

**Keywords:** amide, biological activity, DFT, heterogeneous catalyst, homogeneous, hydration of nitrile, mechanism, transition metal

## Abstract

The synthesis of amide building blocks is crucial for producing diverse amide‐containing compounds such as peptides, proteins, and amino acids. The demand for innovative methods of amide bond derivatives, considered as vital nonclassical bioisosteres, is steadily increasing because of its pivotal role in drug development. A highly cost‐effective and efficient approach for generating amide functional groups involves the metal‐catalyzed hydration of nitriles, offering profound implications for both academic and industrial sectors. This review explores the recent successful catalytic systems, encompassing both homogeneous and heterogeneous solid catalysts that enhance the catalytic transformation of nitriles into amides. Furthermore, theoretical studies employing density functional theory calculations to elucidate the cooperative mechanism between the catalyst and the carbon–nitrogen bond in nitriles are overviewed.

## Graphical abstract

Amide vital functional group plays a key role in the composition of amide‐containing compounds, including peptides, proteins, and amino acids. Demand for innovative amide derivatives grows due to their crucial role in drug development. A cost‐effective method uses metal‐catalyzed hydration of nitriles to amides. This review covers the most recent homogeneous and heterogeneous catalytic systems in the hydration of nitriles to amides, with an overview on their mechanistic aspects.

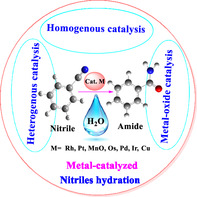

## 1 Introduction

**FIGURE 1:**
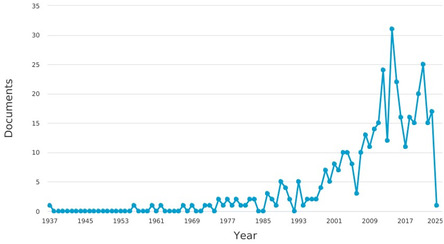
Temporal evolution of the number of documents published per year. *Source* *:* Science Direct.

**FIGURE 2:**
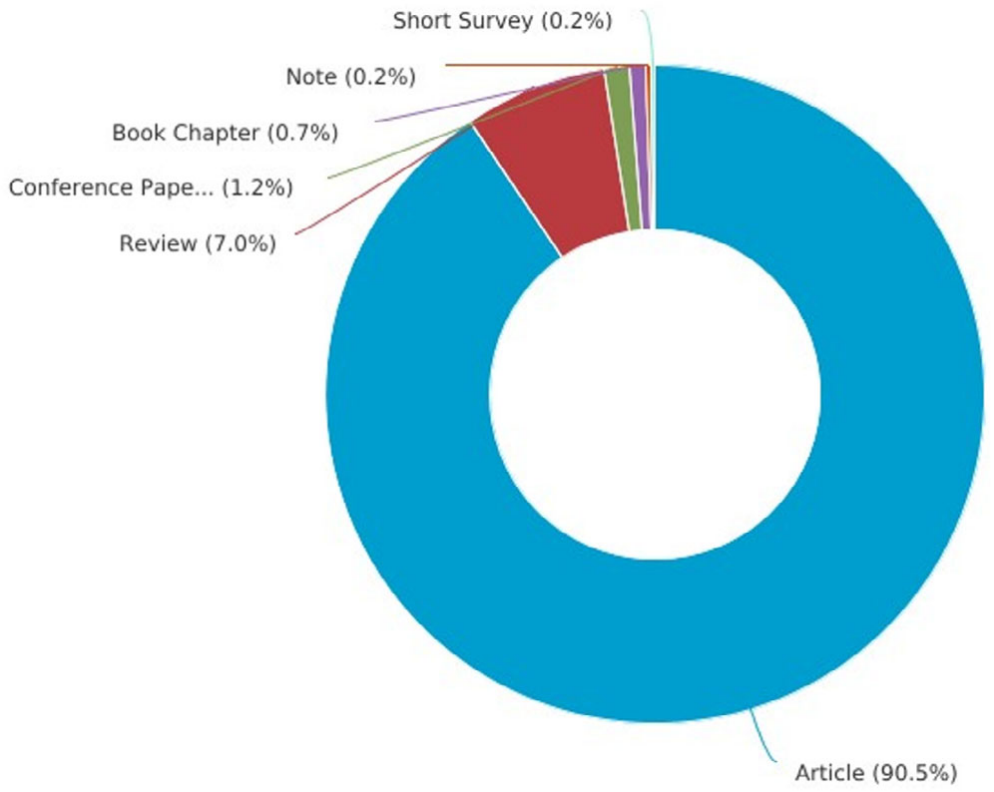
Percentage distribution of the type of published documents. *Source* *:* Science Direct.

The hydration reaction of a nitrile to the corresponding amide is a classic reaction that has garnered significant interest [1]. Historically, the number of articles published on this reaction was restricted from 1885 to 1993, with fewer than five per year focusing on the progression of this reaction. However, between 2009 and 2017, more than a dozen papers have been published annually on this synthetic route, reaching up to 30 articles per year. The increasing number of research reports and articles published in recent years reflects the escalating interest and attention from contemporary researchers toward this reaction, as illustrated in Figure 1. In this regard, research articles dominate the publications at 90%, followed by reviews at 7%, conference papers at 1.2%, book chapters at 0.7%, notes at 0.2%, and short surveys at 0.2% (Figure 2).

Metal‐catalyzed hydration of nitriles is an economically advantageous and atom‐efficient route for synthesizing amide compounds [2, 3–4]. This transformation holds paramount importance in synthetic organic chemistry as the resulting amides have a wide range of applications [5, 6, 7–8]. Furthermore, the amide bond is crucial and of great interest in the pharmaceutical sector due to its significant biological activity [9, 10, 11–12], including but not limited to anticancer, antiviral, antidepressant, antipsychotic, anti‐inflammatory, and antibacterial effects [13, 14, 15, 16–17]. Additionally, the amide is a key constituent of proteins, peptides, and amino acids [18, 19, 20–21].

**SCHEME 1:**
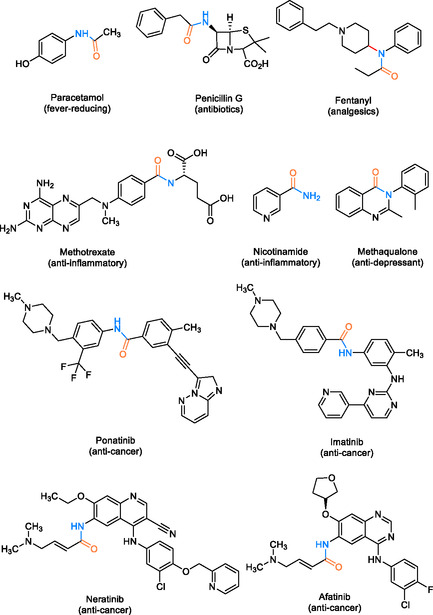
Examples of biologically active molecules as pharmaceutical drugs.

The widespread utilization of paracetamol highlights the importance of understanding the biological effects of amides in drug development and therapeutic practices [22]. Apart from paracetamol, amide linkages are prevalent in various important drug classes, including antibiotics like penicillin G [23], analgesics such as fentanyl [15], anti‐inflammatories like methotrexate [24], and nicotinamide [25], as well as anticancer agents like imatinib [1], neratinib, ponatinib, and afatinib [24]. Additionally, certain amides exhibit antidepressant properties, such as methaqualone [25]. Among the hybrids shown in Scheme 1, some are commercially available, while others are currently under clinical investigation.

Traditionally, the hydration reaction of nitrile using acid or a strong base‐mediated method was the conventional approach for producing amide compounds [26, 27]. However, this method encountered several challenges, including over‐hydrolysis leading to the formation of carboxylic acids, the generation of unwanted by‐products, the complexity of product separation from the reaction mixture, and the requirement for precise control over reaction conditions. Some chemists have strived to develop an efficient biocatalyst system to overcome these challenges and enhance the yields of amide synthesis [28]. The nitrile hydratase (NHase) enzyme has emerged as a promising candidate for facilitating the selective hydration of nitriles into amides under mild conditions [29, 30, 31–32]. However, the widespread adoption of this protocol has been hindered by the specific handling requirements of microorganisms, their high cost, and their limited substrate specificity [33]. Moreover, a selected number of metal‐free catalysts for hydration of nitriles, including ionic and biological catalysts, exhibited low activation barriers and enhanced activity, resulting in more favorable amide yields. Examples include NaOH in NH_3_·H_2_O–DMSO [26], trifluoromethanesulfonic acid [34], the water extract of red mud (WERM)/H_2_O_2_ [35], choline hydroxide [4], and water extract of banana peel ash (WEB) using H_2_O_2_ [36]. However, all these methods have limitations, such as the need for oxygenated water, bases, or the use of organic solvents.

Recently, transition metal catalysts have emerged as efficient tools for activating the cyano group using water as a nucleophile for nitrile hydration. These catalysts are known for their efficiency, environmental friendliness, easy isolation from the reaction medium [37, 38–39], and the ability to be reused multiple times without compromising their reactivity. Various types of catalysts, including homogeneous [40, 41], heterogeneous [1, 42], and metal oxide [43, 44] catalysts, have been utilized to enhance the reactivity of diverse nitriles in the presence of water. This review offers a comprehensive analysis of many metal‐based catalytic systems used in nitrile hydration, including both homogeneous and heterogeneous catalytic systems. The discussion covers solid inorganic/organic hybrid support and metal oxide catalysts. Computational studies focusing on the reactivity and mechanism of nitrile hydration using density functional theory (DFT), as documented in related literature, were discussed.

## 2 Base Catalysts

**SCHEME 2:**
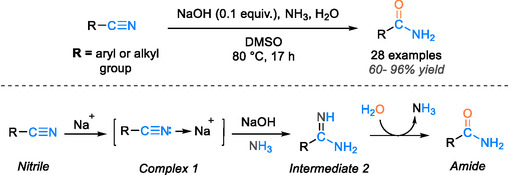
Proposed base‐catalyzed hydration reaction mechanism using NaOH.

The base‐catalyzed transformation of nitrile into amide represents the conventional approach in this hydration process. However, it may sometimes lead to overhydration, resulting in the formation of carboxylic acids or the production of unwanted by‐products. To address these challenges, an alternative reaction pathway uses organic solvents to control the process and halt hydration at the amide formation stage [45]. Chen et al. achieved high efficiency (68%–96% yields) in hydrating 39 nitriles using CsOH in DMSO across ambient to 100°C (1–36 h) [46]. In contrast, Schmid et al. [47] used NaOH in an EtOH/H2O (7:3) solvent at 90°C for 17 h, delivering 60%–95% yields. In 2020, Wang et al. [26] employed NaOH with NH3·H2O–DMSO under mild conditions (80°C, 7 h) to hydrate 28 nitriles to amides, yielding 60%–96% and proposing a mechanism involving Na^+^ activation and ammonia addition (Scheme 2). Initially, the activation of Na^+^ ions in NaOH can lead to coordination with nitrile, resulting in complex 1. Subsequently, complex 1 undergoes the formation of an intermediate. Facilitated by a selective nucleophilic addition reaction with ammonia, intermediate 2 undergoes further hydration to yield the desired amide (Scheme 2). Nevertheless, a drawback of these approaches is their slow reaction kinetics that necessitate careful control over the base loading when utilizing organic solvents. Challenges arise during separation and purification due to the stubborn nature of these solvents, demanding high‐pressure processes and resulting in elevated energy consumption. Moreover, the base‐catalyzed approach mandates the use of chemical additives to enhance nucleophilic attack. Hence, this review will emphasize metal‐catalyzed systems as a promising alternative to address these limitations.

## 3 Mineral Residue‐Based Catalyst

**SCHEME 3:**
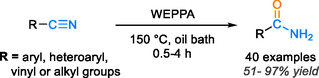
Nitrile hydration is catalyzed by WEPPA.

A recent environmentally conscious approach has emerged for the hydration of nitriles into amides, using agricultural or bioorganic waste to yield highly satisfactory results in amide compound synthesis under eco‐friendly conditions. In this regard, the efficacy of employing water extract from pomelo peel ash (WEPPA), sourced from agricultural waste ash, was investigated in the transformation of nitriles into amides via hydration reaction. This method represents a sustainable and environmentally friendly approach for achieving the desired transformation as depicted in Scheme 3. The utilization of such an extract, as a source of the base that catalyzes the hydration reaction, resulted in substantial amide functional group yields ranging from 51% to 97% within a timeframe of 0.5–4 h at 150°C, with the possibility to reuse WEPPA for up to four cycles [48].

**SCHEME 4:**
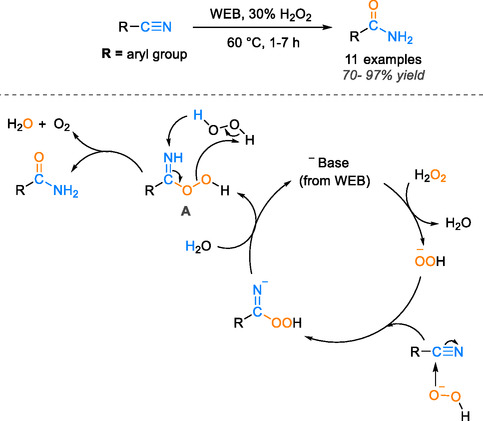
Proposed mechanism for aryl nitrile hydration to amide catalyzed by the WEB.

In a separate investigation, Das et al. utilized a solution containing 30% hydrogen peroxide (H_2_O_2_) in an extract derived from agricultural waste, referred to as WEB. This innovative approach yielded highly favorable results in the conversion of aromatic nitriles to amides, with impressive yields ranging from 70% to 97% at a consistent temperature of 60°C during 1–7 h [36]. The proposed mechanism of this reaction, shown in Scheme 4, involves the WEB rendering hydrogen peroxide alkaline, thereby facilitating the smooth progression of the reaction. Initially, the base from WEB deprotonates H_2_O_2_, giving rise to the HOO^−^ ion. Subsequently, this ion attacks the carbon nitrile, leading to the formation of the peroxycarboximidic acid intermediate (A). Finally, intermediate A reacts with another molecule of H_2_O_2_, culminating in the formation of the desired amide product [36]. Although utilizing the water extract of agro‐waste ash presents a sustainable and environmentally friendly approach, it does have certain limitations when compared to transition metal‐based catalysts. Metal catalysts provide advantages such as higher catalytic efficiency, a wider range of applicable substrates, the potential for regeneration, and superior performance under various reaction conditions (*vide‐infra*).

## 4 Homogenous Catalysts

**SCHEME 5:**
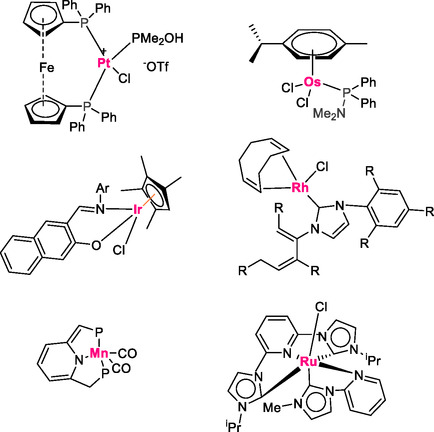
Complexes catalyzed hydration of organonitriles into amides.

**SCHEME 6:**
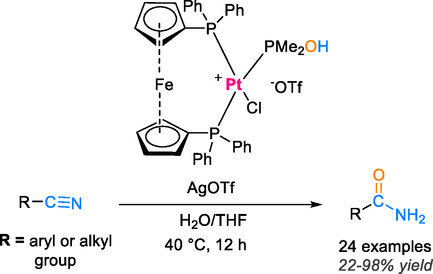
Nitrile hydration catalyzed by [1,1'‐ferrocenendiyl bis(diphenylphosphine)]PtCl_2_ and PMe_2_OH.

Homogeneous catalysis uses catalysts that share the same phase as the reactants, typically dissolved in solution. These catalysts can offer high selectivity and uniform interaction with substrates, making them especially effective for demanding nitrile substrates. A common limitation, however, is the difficulty of separating and recovering the homogeneous catalyst after the reaction. In transition‐metal–based homogeneous systems for converting nitriles to amides, auxiliary organic ligands are often employed. These ligands play a crucial role by modulating the electronic and steric environment of the metal center, thereby influencing the nitrile hydration pathway and facilitating efficient amide bond formation [41, 49, 50]. The most effective homogeneous catalytic systems that have been utilized in nitrile hydration reactions contain Pt, Os, Rh, Mn, Ir, and Ru metal ions (see Scheme 5). In a seminal work, Xing et al. have introduced a novel donor–acceptor strategy to enhance the catalytic efficiency of the platinum complex [1,1'‐ferrocenendiyl‐bis(diphenylphosphine)]PtCl_2_, PMe_2_OH, and AgOTf in the hydration reaction of various nitriles using water [51]. This innovative approach involves the incorporation of a bidentate ligand with both electron‐rich and electron‐deficient groups. The platinum catalyst adopts a square planar geometry, with the electron‐rich diphenylphosphine and electron‐accepting dimethylphosphine ligands in a *trans* configuration, facilitating the nucleophilic attack of the hydroxyl group of the PMe_2_OH ligand on the electrophilic carbon of the nitrile and hydroxynitrile groups. The primary objective is to enhance the activation of the cyanide group and promote nucleophilic attack. Consequently, the synthesis of amide groups was achieved with high yields ranging from 22% to 98% at 40°C over a period of 12 h (Scheme 6).

**SCHEME 7:**
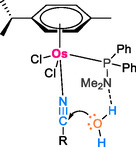
Cooperative effect of amino‐phosphane ligand PPh_2_(NMe_2_).

González‐Fernández et al. [52] have introduced a more effective complex involving the formation of an osmium complex [OsCl_2_(η6‐p‐cymene){PPh_2_(NMe_2_)}] using the amino‐phosphane PPh_2_(NMe_2_) ligand. This excellent approach aims to enhance the catalytic activity in the hydration reaction of nitrile compounds in water without the need for added base or acid, maintaining a neutral reaction medium. The outcomes are particularly promising, as osmium demonstrates increased activity, resulting in remarkable yields of 69%–96%, significantly surpassing previous results, and producing amide compounds within a relatively short timeframe of 0.5–24 h at temperatures ranging from 80°C to 100°C under inert atmosphere. The amino‐phosphane ligand PPh_2_(NMe_2_) facilitates the coordination of the nitrile group with osmium, enabling the amino substrate to establish a hydrogen bond with the water molecule. Subsequently, the carbon undergoes nucleophilic attack by water, leading to the formation of the amide product (to Scheme 7).

**SCHEME 8:**
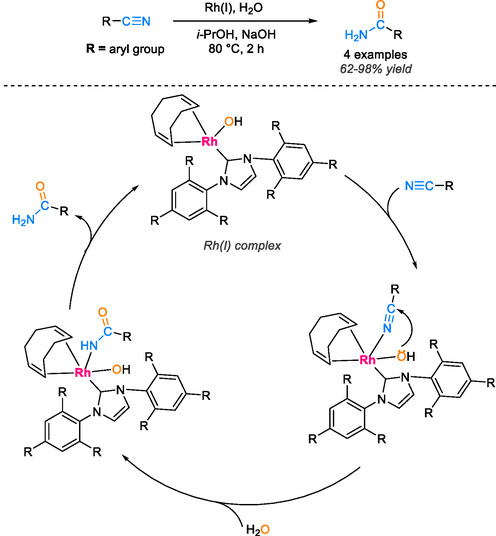
Catalytic hydration of nitriles using Rh(I)‐N‐heterocyclic complexes.

Recently, Czégéni and coworkers reported the transformation of organonitrile substrates into their corresponding amides using Rh(I)‐N‐heterocyclic complexes in a water/isopropanol (1:1 v/v) solvent mixture under ambient conditions. In this process, NaOH was employed as a base to facilitate nucleophilic attack with hydroxide ions through coordination to the Rh(I) complex, activating the carbon of the nitrile substrate at 80°C for 2 h [53]. The reaction yielded an impressive 98% yield in the case of benzonitrile substrate. The proposed reaction mechanism involves the nucleophilic attack of a Rh(I)‐coordinated HO^−^ ion on the nitrile carbon atom, which is activated by the coordination of the nitrile group to the metal ion via the nitrogen atom. The presence of hydroxide ions from NaOH serves to promote and accelerate the formation of the amide products (Scheme 8).

**SCHEME 9:**
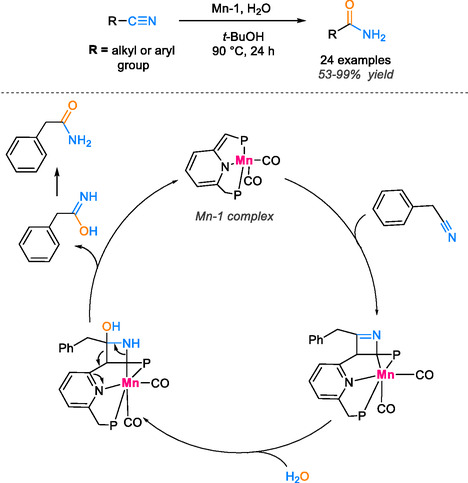
Catalytic hydration of nitriles using manganese pincer complex.

The use of manganese pincer complex [54] presents an innovative approach to activating double and triple bonds, characterized by the simultaneous binding of the substrate to both ligand and metal center. This catalytic system was employed in the hydration reaction of phenyl acetonitrile. The activation of the nitrile group was achieved through the formation of a bond between the carbon of the ligand and the carbon of the cyanide group. In addition, the establishment of bonds between manganese and the nitrogen of the nitrile group results in the formation of the enamido complex. Subsequent transformation of the intermediate involves nucleophilic attack by water as the solvent, inducing tautomerization and formation of amide functional groups in high yields ranging from 53% to 99% after 24 h at 90°C (Scheme 9).

**SCHEME 10:**
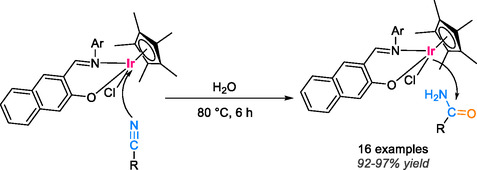
Nitrile hydration to amides catalyzed by half‐sandwich iridium complex.

Half‐sandwich iridium complexes, featuring *N*,*O*‐chelate Schiff base ligands with nitrogen and oxygen donor coordinating atoms, serve to stabilize the complex and enhance its catalytic activity significantly [55]. These complexes have been successfully applied in the hydration of aryl nitrile derivatives bearing both electron‐donating and electron‐withdrawing groups, resulting in highly favorable outcomes in aqueous environments without the need for additional base or acid. The soluble aqueous complex exhibits remarkable catalytic performance in the synthesis of amides, delivering impressive yields of 92%–97% within just 6 h at 80°C with a minimal 0.5 mol% catalyst loading under mild reaction conditions (Scheme 10).

**SCHEME 11:**
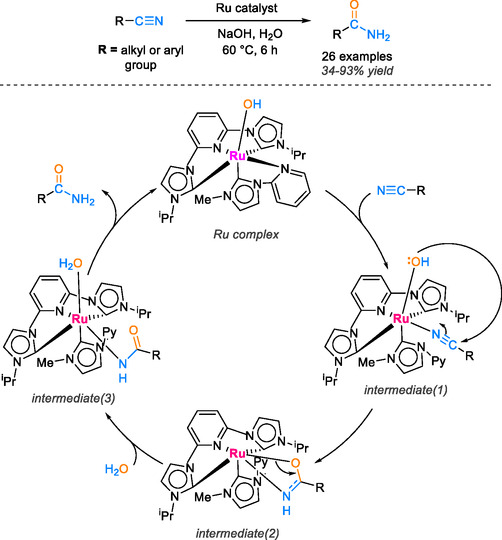
Proposed mechanism of nitrile hydration catalyzed by ruthenium complex.

Singh and collaborators have very recently pioneered the development of a series of electron‐rich ruthenium complexes, each complexed with distinct *N*‐heterocyclic carbene ligands, for the efficient hydration of a wide range of aromatic and heterocyclic nitriles into their corresponding amide counterparts [56]. The results obtained from these studies are highly promising, achieving yields of up to 93% at 80°C over a 24 h period. Notably, this homogeneous catalyst demonstrated exceptional recyclability, being successfully recovered and reused five times. Following each catalytic cycle, the reaction mixture was cooled to 4°C for 12 h, resulting in the crystallization of the amide product. The aqueous solution containing the ruthenium complex was then reused for a total of five cycles. In the mechanistic investigation, spectroscopic analyses were employed to validate the formation of crucial reaction intermediates during the catalytic hydration of nitriles by the ruthenium complex. Initially, the complex underwent a base‐induced attack, leading to the formation of a hydroxyl bond with the ruthenium metal, thereby enhancing nucleophilic attack capabilities. Subsequently, the coordination of the nitrile group facilitated the generation of an intermediate species (1) as shown in Scheme 11.

**SCHEME 12:**
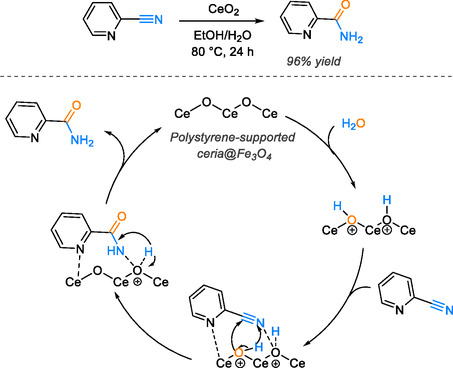
Proposed mechanism of nitrile hydration catalyzed by CeO_2_‐polystyrene‐Fe_3_O_4_.

The cyano carbon was then subjected to a nucleophilic attack by the hydroxyl group, resulting in the formation of an intermediary species (2) featuring an iminolate ligand. Furthermore, a water molecule bound to the ruthenium complex, causing the iminolate group to revert to an amido form, giving rise to an intermediate species (3). Finally, intermediate (3) underwent protonolysis, culminating in the production of the amide product and the regeneration of the catalyst, thereby completing a full catalytic cycle (Scheme 12).

A comparison of the reported catalytic systems is provided (see Table 1). Notably, many of these methods exhibit several drawbacks, such as the need for additional ligands, large base loadings, hazardous organic solvents, and an inert atmosphere. In contrast, the catalysts described here offer high selectivity, lower catalyst loadings, and impressive yields. Nevertheless, practical applications are sometimes limited due to difficulties in catalyst recovery and recyclability, which are advantages of heterogeneous systems.

**TABLE 1:** Comparison of metal‐based homogenous catalytic systems for the hydration of nitriles into amides.

Catalyst	Ligand	Base	Cat. loading (mol%)	Temperature (°C)	Time (h)	Solvent	Yield (%)	Ref
Pt	1,1′‐Ferrocenendiyl bis(diphenylphosphine)	–	0.1–0.5	40	12	H_2_O/THF	22–98	[51]
Os	PPh_2_(NMe_2_)	–	1	80–100	0.5–24	water	69–96	[52]
Rh	*N*‐heterocyclic carbene	NaOH	1	80	2	iPrOH	98	[53]
Mn	N,P‐pincer	–	0.5	90	24	t‐BuOH	53–99	[54]
Ir	*N*,*O*‐chelate Schiff base	–	0.5	80	6	water	92–97	[55]
Ru	*N*‐heterocyclic carbene	NaOH	1	80	24	water	34–93	[56]

## 5 Heterogeneous Catalysts

Heterogeneous catalysts are generally more suitable for large‐scale applications due to their stability and recyclability. While their intrinsic selectivity may be slightly higher than that of homogeneous systems, thoughtful design of the support and optimization of reaction conditions can still achieve high yields, even for challenging substrates. This versatility enables researchers to tailor catalysts to specific nitrile substrates and desired reaction environments. A range of heterogeneous metal‐based catalysts has been developed for nitrile hydration, including supported metal nanoparticles, magnetic catalysts, metal oxides, and hybrid systems. Each type offers distinct advantages: For instance, magnetic catalysts allow easy recovery with an external magnetic field, whereas supported nanoparticles typically provide high surface area and enhanced reactivity.

### 5.1 Organic Polymer‐Supported Metal Catalyst

Organic polymers play a crucial role as organic carriers in heterogeneous catalysis, finding widespread applications [57, 58–59]. They serve to stabilize transition metals, introduce surface organic functions that enhance chemical transformations, and provide highly flexible supports that improve reagent accessibility, ultimately leading to enhanced catalytic efficacy. In a recent study, Muñoz‐Espi and coworkers utilized the potential of polystyrene to support ceria oxide in the hydration of 2‐cyanopyridine to 2‐picolinamide [60]. By integrating ceria‐polystyrene materials, they improved reusability of the catalyst and simplified its recovery. Notably, this catalyst can be effortlessly dispersed in water or ethanol as solvents of the hydration reaction. The catalytic system exhibited nearly complete conversion of nitrile compounds to amide compounds, with yields approaching 100% after 6–8 h at 80°C. Furthermore, it was easily recovered by centrifugation and could be reused up to five times without any discernible loss in catalytic efficiency. In line with the use of cerium(IV) oxide for hydration of nitriles into amides, Muñoz‐Espi devised a new catalytic system that integrates a magneto responsive core to streamline the recovery process of the polystyrene‐supported catalyst [61]. This practical approach utilizing an external magnetic field for catalyst retrieval led to heightening the catalytic efficiency of the ceria nanoparticles. The use of nanoparticles has proven to be highly effective, yielding impressive results in the synthesis of 2‐picolinamide, with conversion rates of up to 96% at 80°C within a 24 h timeframe. Noteworthy is the catalyst's ability to be reused up to four times that demonstrates its sustainability. The proposed reactivity mechanism, as depicted in Scheme 12, elucidates the interaction between the substrate and the magnetic ceria catalyst supported on polystyrene. The process begins with the coordination of the nitrile group with cerium, followed by the dissociation of a water molecule. Subsequently, there is a sequential transfer of oxygen and protons from water to the nitrile group, facilitated by a series of rapid molecular rearrangements. The final step ultimately results in the production of the amide product.

### 5.2 Magnetic Materials‐Supported Metal Catalyst

The utilization of magnetic support in catalysis has revolutionized the efficiency and effectiveness of catalytic systems [62, 63], particularly in the hydration of nitrile compounds into amide groups. This innovative approach has endowed the catalyst with a myriad of desirable properties, including high efficiency, ease of recovery, and reusability [64, 65]. Notably, Kazemi Miraki and coworkers [66] have developed a cutting‐edge catalyst system where catalyst is immobilized on silica and magnetic iron and copper coordinated with an N‐heterocyclic carbene ligand. This unique system exhibits heterogeneous, mesoporous, and paramagnetic properties that not only facilitate the activation of the cyanide group but also enable the catalyst to be reused in subsequent cycles.

**SCHEME 13:**
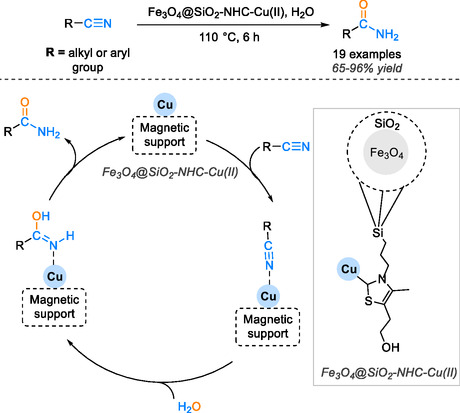
Proposed mechanism of nitrile hydration catalyzed by Fe_3_O_4_@SiO_2_‐NHC‐Cu(II).

The application of this magnetic catalyst in the hydration of various aromatic benzonitrile derivatives in water as a solvent has yielded remarkable results, with high yields ranging from 65% to 96% achieved in less than 8 h at 110°C. The magnetic nature of the catalyst allows for effortless retrieval using an external magnet, enabling its reuse for up to six cycles without a significant decline in catalytic efficacy. In exploring the mechanism of this catalytic cycle, the initial coordination of the nitrile with copper triggers the activation of the nitrile group through water acting as a nucleophile, leading to the formation of a hydroxyimine reaction intermediate. Subsequent tautomerization of the intermediate results in the production of the final amide product, as illustrated in Scheme 13.

**SCHEME 14:**
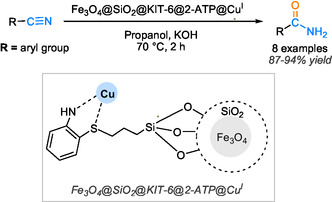
Nitrile hydration catalyzed by Fe_3_O_4_@SiO_2_@KTP‐6@2‐ATP@Cu^I^.

This detailed insight into the catalytic pathway underscores the efficiency and precision of this magnetic catalyst system in driving complex organic transformations. In a recent study, Moradi and Ghorbani‐Choghamarani introduced a novel catalytic system utilizing copper(I)‐supported magnetic mesoporous silica for the hydration reaction [67]. This catalyst demonstrated remarkable catalytic efficiency, leading to excellent yields of amide products ranging from 87% to 94% within 2 h at 70°C, with the assistance of KOH and isopropanol as the solvent in the reaction mixture. The magnetic nature of the catalyst allows its easy separation from the reaction medium using an external magnet, then facilitating its reuse in multiple successive runs for up to six cycles, as illustrated in Scheme 14.

### 5.3 Titan‐Supported Metal Catalyst

**SCHEME 15:**
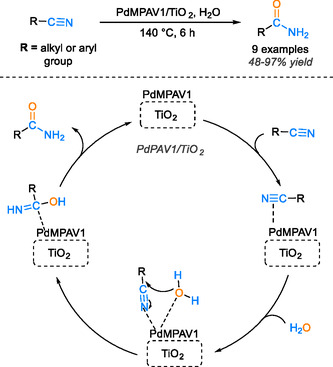
Proposed mechanism of nitrile hydration catalyzed by PdMPAV_1_/TiO_2_.

Titanium dioxide (TiO_2_) [68, 69] is a widely utilized support in heterogeneous catalysts due to its exceptional stability and mesoporous surface characteristics [70, 71]. This choice effectively mitigates the problem of particle agglomeration, which can obstruct the active sites within the catalyst, consequently reducing the efficiency and activity of metal‐catalyzed reactions. The titanium support exhibits a strong affinity with metals, showcasing remarkable chemical stability and distinctive acid–base properties. In a significant development by Srinivasa Rao and colleagues, a catalyst has been devised for the hydration of nitriles to amides [72]. This catalyst is prepared on a foundation of titania‐supported palladium‐exchanged vanadium‐incorporated molybdophosphoric acid catalysts. The majority of these catalysts, known as PdMPAV_1_, exhibit remarkable uniformity under the prevailing reaction conditions. By anchoring them on titania, these bulk catalysts are transformed into efficient heterogeneous catalysts. The selection of titania as the support is underpinned by its ability to maintain the structural integrity of the Keggin unit even at elevated temperatures, a quality lacking in its unsupported counterpart. A pivotal attribute of the titania support is its capacity to release oxygen from the surface, facilitated by lattice oxygen mobility, and to create oxygen vacancies under relatively mild conditions. This intrinsic cleanliness enhances nitrile activation and concurrently facilitates the adsorption of water molecules onto the catalyst surface. The activation of the nitrile bond is orchestrated by the palladium sites, prompting a nucleophilic attack by hydroxyl ions from water, leading to the formation of the reaction intermediate, imine. Subsequent tautomerization of the imine yields the final amide product. This detailed reaction mechanism is elucidated in Scheme 15.

Remarkably, this catalyst has demonstrated the ability to forge amide bonds in water sans the use of organic solvents, delivering yields ranging from 48% to 97% at 140°C over a span of 6 h. Furthermore, it showcases reusability, retaining its catalytic efficiency after being reused thrice.

### 5.4 Silica‐Supported Metal Catalyst

**SCHEME 16:**
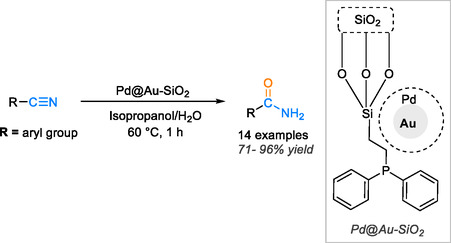
Hydration of nitriles to amides catalyzed by Pd@Au‐SiO_2_.

Silicon dioxide (SiO_2_), commonly known as silica, stands out as a remarkable choice for supporting heterogeneous catalysis due to its unique metal‐stabilizing properties, high specific surface area, substantial pore volume, and medium‐sized pores. A catalyst anchored on silica can undergo multiple cycles of reuse, showcasing its sustainability and cost‐effectiveness [73, 74]. In line with this, Kalita and colleagues have developed a highly efficient bimetallic Au–Pd catalyst supported on silica for the hydration of organonitriles into amide compounds [75]. This new approach harnesses the synergistic effects of the bimetallic core–shell palladium–gold system, which exhibits exceptional catalytic efficiency in organic syntheses. By leveraging silica as a catalytic support, the heterogeneity and specific surface properties of the catalyst are significantly enhanced. The application of this catalytic system in a model nitrile hydration reaction, conducted in a water/isopropanol (1:1 v/v) solvent mixture at 60°C, yielded outstanding catalytic performance within a short duration of 1 h, resulting in impressive yields ranging from 71% to 96% (Scheme 16).

It can be utilized for successive cycles, maintaining consistent catalytic efficiency for up to five cycles without a significant loss. Efficient recovery and reuse of the heterogeneous catalyst supported on silica are facilitated by separation techniques such as centrifugation and filtration, followed by a thorough washing with water/isopropanol (1:1 v/v) and drying process. This streamlined approach ensures the sustainable utilization of the catalyst, underscoring its practicality and environmental friendliness in catalytic applications.

### 5.5 Carbon‐Supported Metal Catalyst

The use of carbon as a support in heterogeneous catalysis is paramount due to its wide array of physicochemical properties [76, 77], including exceptional thermal stability and a significant surface area.

**SCHEME 17:**
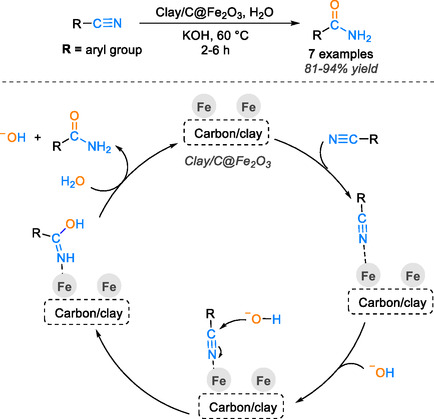
Proposed mechanism of nitrile hydration catalyzed by clay/C@Fe_2_O_3_.

The key to achieving a well‐dispersed and active catalyst that can be easily recovered and reused multiple times lies in the presence of a substantial surface area characterized by accessible pores and high adsorption capacity. Carbon surfaces are rich in various heteroatoms such as nitrogen, oxygen, and hydrogen, which are strategically incorporated to introduce functional groups. These functional groups impart acidic, basic, or hydrophilic properties to the surface, enhancing its catalytic performance. In this context, iron oxide supported on an activated carbon/clay surface has been employed in the hydration reaction of diverse nitrile groups in water [78]. This catalyst exhibited exceptionally high catalytic efficiency with a low amount of iron (0.05 mol%) at 60°C within 6 h, in the presence of KOH under aerobic conditions, yielding results between 81% and 94%. Furthermore, the activated clay/carbon surface enhances the stability of the iron metal and catalytic efficiency, enabling the catalyst to be utilized for up to six cycles without any discernible loss in efficiency. The investigation into the catalytic cycle mechanism elucidated the coordination of the cyano bond with iron, followed by the nucleophilic attachment of the electrophilic carbon by hydroxyl ions from the KOH base, leading to the formation of the reaction intermediate 1 hydroxyl imine. Subsequent interaction with a water molecule culminates in the generation of the final amide product (see Scheme 17), showcasing the intricate yet efficient pathway through which this catalytic system operates.

### 5.6 Solid Metal Oxide Catalyst

**SCHEME 18:**
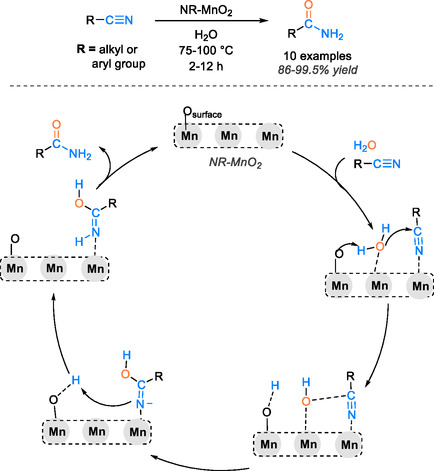
Proposed mechanism of nitrile hydration catalyzed with NR‐MnO_2_.

Transition metal oxides are among the most crucial materials in heterogeneous catalysts for diverse chemical transformations [79]. In the hydration reaction of nitriles to amides, the metal oxide plays a crucial role in activating the nitrile group. This is attributed to the oxygen‐rich surface, which expedites the rapid coordination of the substrate with the metal and facilitates the nucleophilic attack of water. Additionally, the formation of hydrogen bonds with the metal oxide surface further enhances this process. In this context, we will delve into the efficacy of key metal oxide catalytic systems, specifically MnO_2_, Ru/MnO_2_, and Ag/MnO_6_, as documented in the literature for the hydration reaction of nitriles. Hai Wang and collaborators harnessed a selective and highly efficient manganese oxide‐based catalytic system for the synthesis of amide groups [44]. Indeed, this catalyst has exhibited remarkable catalytic efficiency, yielding up to 99.5% conversion of various nitrile derivatives within a timeframe of 2–12 h at 75°C. The NR‐MnO_2_ catalyst consistently maintained stable catalytic performance over five consecutive recycling runs. The outstanding catalytic prowess of MnO_2_ can be attributed to the presence of active surface oxygen species, which promote the generation of abundant hydroxyl species that facilitate the nucleophilic attack on the nitrile group, a critical step in the hydration process. The active oxygen sites on MnO_2_ effectively initiate the activation of water molecules through hydrogen bonding between the metal oxide and water (Mn…O…H–OH), leading to the generation of nucleophilic hydroxyl species (OH) and metal oxide hydrogen‐bonded species Mn…O…H. The hydroxyl species are inclined to target the nitrile group. Then, the nitrogen atom pulls out the necessary hydrogen proton on Mn…O…H. Concurrently, the O site on the oxide surface is regenerated, leading to the generation of the amide product. This catalytic cycle mechanism is depicted in Scheme 18.

**SCHEME 19:**
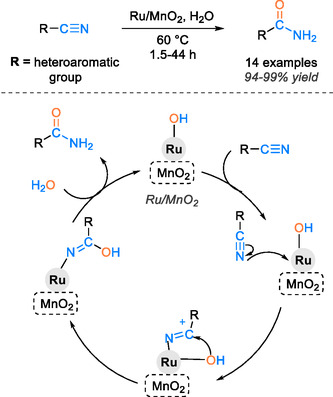
Proposed mechanism of nitrile hydration catalyzed with Ru/MnO_2_.

Hussain and coworkers have developed a catalytic system utilizing manganese metal oxide as support for the ruthenium catalyst [80]. The heterogeneous catalyst demonstrates high catalytic efficiency for the hydration of diverse aliphatic, aromatic, and heteroaromatic nitriles in water as a reaction solvent at 60°C, attaining conversions ranging from 80% to 99% within 5–23 h. Furthermore, the catalyst can be reused up to five times without any loss of catalytic efficiency. The proposed mechanism for this study primarily revolves around the nucleophilic attack of the nitrogen atom in the nitrile group on ruthenium, leading to the formation of a carbocation. This is followed by the nucleophilic attack of the hydroxyl group on the carbon of the nitrile, resulting in the formation of an iminolate intermediate. Subsequently, the amide product is formed, and the regeneration of the ruthenium‐hydroxide species occurs through ligand exchange between the complex iminolate and water (Scheme 19).

**SCHEME 20:**
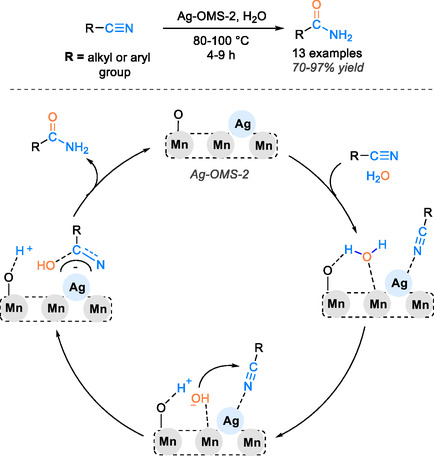
Catalytic cycle of nitrile hydration to amide catalyzed with Ag‐OMS‐2.

Kalimani and Khorshidi have introduced a high‐performance metal oxide octahedral structure incorporating silver within manganese oxide for the hydration of 13 nitriles into the corresponding amides in water [42]. This intriguing approach boasts several notable features, including the catalyst's exceptional thermal stability, porous structure, ease of recycling and work‐up, and the environmentally friendly nature of the process, due to the utilization of water as both a solvent and a hydrating agent. This catalyst yielded satisfactory results, achieving a yield range of 70%–97% at temperatures between 80°C and 100°C within a short reaction time of 4–9 h. Furthermore, the catalyst exhibited remarkable recyclability, showing only a slight decrease in efficiency after six consecutive cycles. A potential reaction mechanism for nitrile hydration on Ag‐OMS‐2 is illustrated in Scheme 20.

Initially, the nitrile coordinates through nitrogen interaction with the surface of positively charged silver nanoparticles. The active oxygen sites of the octahedral manganese oxide structure acting as Brønsted bases efficiently activate water molecules via Mn/O–H–OH interactions, leading to the formation of hydroxyl species. Subsequently, there is a nucleophilic addition of hydroxyl to the nitrile. After nitrogen facilitates the extraction of hydrogen from Mn–O/H, active oxygen sites regenerate on the metal oxide. Ultimately, the resulting hydrated nitrile species undergo ligand exchange with the solvent, resulting in the production of final amide product (Scheme 20).

Akansha Mehta et al. [81] evaluate the reactivity of m‐MnO_2_ for converting nitriles to amides under visible light in weakly basic and neutral media, with products isolated and confirmed as amides by GC‐MS and ^13^C NMR. Among three photocatalysts (c‐MnO_2_, s‐MnO_2_, and m‐MnO_2_), m‐MnO_2_ delivers the highest yields (70%–90%) over 5 h in water and 10 h in Et_3_N as solvent, while c‐MnO_2_ and s‐MnO_2_ provide ∼50% yields, as evidenced by GC‐MS and ^13^C NMR validation of amide formation. The catalyst also demonstrates robust reusability, maintaining performance over at least five cycles and can be easily separated by centrifugation.

To demonstrate the efficiency of these catalytic systems, a comparison of the catalytic activity of the heterogeneous catalysts is summarized in Table 2. The catalytic activity of these heterogeneous catalysts enables the generation of excellent yields of amide derivatives using low catalyst loadings in water as the solvent, at various temperatures, and under open‐air conditions. Furthermore, the separation of the final amide product can be easily achieved by simple filtration or by using an external magnetic pellet.

**TABLE 2:** Comparison of metal‐based heterogenous catalytic systems for the hydration of nitriles into amides.

Catalyst	Number of run	Base	Cat. loading (mol%)	Temperature (°C)	Time (h)	Solvent	Yield (%)	Ref
CeO_2_‐polystyrene‐Fe_3_O_4_	4	–	5–40 mg	80	24	EtOH/H_2_O	22–98	[61]
PdMPAV_1_/TiO_2_	3	–	80 mg	140	**6**	Water	48–97	[72]
Pd‐Au‐SiO_2_	5	–	Pd: 0.11 Au: 0.09	60	1	iPrOH/H_2_O	71–96	[75]
Clay/C@Fe_2_O_3_	6	KOH	0.05	60	2–6	Water	81–94	[78]
Fe_3_O_4_‐SiO_2_‐NHC‐Cu(II)	6	–	6	110	6	Water	65–96	[66]
Fe_3_O_4_@SiO_2_@KTP‐6@2‐ATP@Cu^I^	6	KOH	40 mg	70	2	Propanol	87–9	[67]
NR‐MnO_2_	5	–	100 mg	75–100	2–12	Water	86–99	[44]
Ru/MnO_2_	5	–	125 mg	60	1.5–44	Water	94–99	[80]
Ag‐OMS‐2	6	–	4.87 (30 mg)	80–100	4–9	Water	**70–97**	[42]

## 6 Computational Insights into the Hydration Mechanism of Nitriles to Amides

### 6.1 Mechanism of Base‐Catalyzed Nitrile Hydration

**SCHEME 21:**
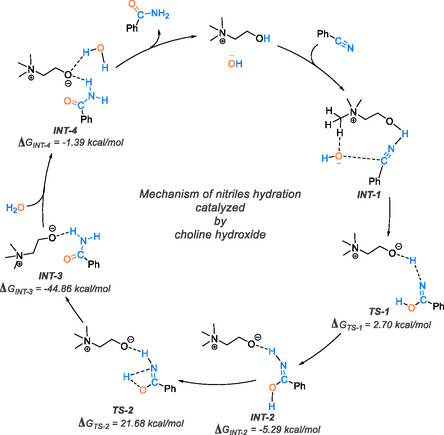
Proposed mechanism of nitrile hydration catalyzed by choline hydroxide using DFT.

DFT analyses were employed to elucidate a plausible reaction mechanism for the transformation of nitrile to amide, providing insights into the reaction pathway and the role of the catalyst. In their study, Choudhury et al. introduced a reaction mechanism employing choline hydroxide as a metal‐free catalytic system [4]. This approach offers the benefit of establishing extra hydrogen bonds with the reactants, thereby facilitating the reaction in aqueous surroundings. The findings substantiate the hypothesis that choline hydroxide (ChOH) reduces the activation energy barrier of the reaction, as determined by DFT calculations, thereby improving the efficiency of the conversion process. The suggested mechanism is outlined in Scheme 21.

Initially, choline hydroxide (ChOH) initiates the reaction by activating the nitrile bond in benzonitrile, leading to the formation of a hydrogen–bonded complex identified as intermediate INT‐1. This interaction was validated using the quantum theory of atoms in molecules. The presence of a hydrogen bond is indicated by the bond critical point (BCP) between the nitrogen of the nitrile group and the hydrogen of the aliphatic OH group in the catalyst, with an electron density at the BCP (ρBCP) ranging from 0.002 to 0.034 atomic units, measuring 0.028 atomic units in this specific case, indicative of a strong hydrogen bond. The initiation of the reaction is gradual and requires heat. After the formation of intermediate INT‐1 in the reaction, a nucleophilic attack by the hydroxide anion on the nitrile carbon occurs. Simultaneously, the activated nitrogen in the nitrile group abstracts a proton from the catalyst, leading to the creation of intermediate INT‐2. This step occurs through the transition state TS‐1, which has an energy barrier of 2.70 kcal/mol. The reduction in the barrier activation is attributed to the stabilization of TS‐1 facilitated by hydrogen bonds. Subsequently, INT‐2 surpasses the transition state TS‐2, encountering a substantial energy barrier of 21.68 kcal/mol in the process of amide formation. The transition to tautomerism is driven by the creation of the considerably more stable intermediate INT‐3. Following this, with the intervention of water, the catalyst is regenerated, and the isolated product is prepared for further analysis.

### 6.2 Mechanism of Metal‐Catalyzed Nitrile Hydration

**SCHEME 22:**
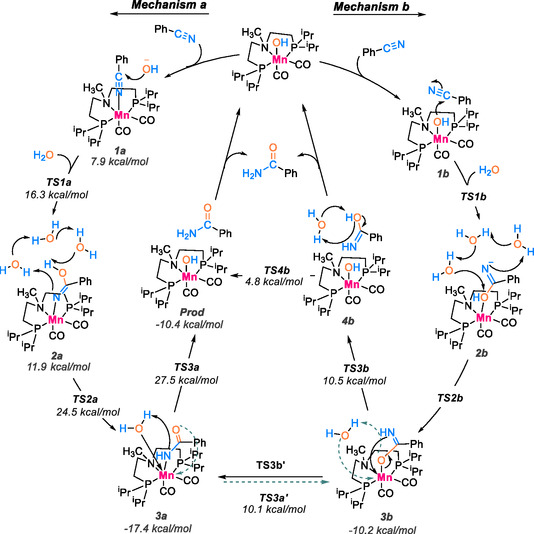
Proposed mechanism of nitrile hydration catalyzed by Mn(N‐CH_3_)OH using DFT.

Taking advantages of DFT in computational investigations proves to be highly beneficial for simulating catalytic reactions, offering a detailed representation of each step in the process [82, 83]. By establishing a comprehensive understanding of the catalytic mechanism, these approaches are increasingly indispensable for predicting and strategically designing more efficient catalysts [84, 85]. This underscores the crucial role of these tools in enhancing the interpretation and justification of experimental findings. In this context, researchers Prejanò and colleagues have proposed two reaction mechanisms for the catalytic cycle involving the hydration reaction of cyanobenzyl into benzamide, catalyzed by the Mn(NCH_3_)‐OH complex [38]. This proposal is substantiated by DFT calculations, and the illustrated catalytic cycle is presented in Scheme 22.

The process begins with the substitution of the hydroxyl group by a nitrile, followed by a nucleophilic attack of the hydroxide on the metal‐coordinated nitrile group within structure **1a**, resulting in the formation of carboximide **2a**. The final step involves tautomerism from carboximide to amide. Calculations confirm an interaction between the carbonyl group and the N‐H group on the PNHP ligand backbone. The intermediate complex **3a** is situated at an unusually low‐energy state, providing a plausible rationale for the formation and isolation of the amide in experimental observations [86]. Subsequent to this, the NH group of the substrate (**3a**) undergoes direct protonation by an external water molecule. Following this, the hydroxide ion from water attacks the Mn ion through TS3a, leading to the regeneration of the initial catalyst and the release of the benzamide product.

In the proposed mechanism b, the metal‐bound hydroxide initiates a nucleophilic attack on a nitrile molecule. This mechanism involves a coordination rearrangement of the substrate, where bonding with the metal shifts from the nitrogen to the carbonyl oxygen, resulting in the intermediate 3b. Nonetheless, the free energy profile calculated for the nucleophilic attack of the Mn‐bound hydroxide on the nitrile substrate is significantly higher (>28.0 kcal/mol) than the profile computed for mechanism **a**. This leads us to rule out the possibility of **3b** being formed through that pathway. The catalyzed process continues with TS3b, where the protonation of the carbonyl oxygen facilitates the substrate‐hydroxide exchange mechanism. In the next step, the iminol is formed and the catalyst is regenerated. The swift tautomerization of iminol leading to the formation of the amide product completes the catalytic cycle.

Babón and coworkers [84] have introduced a catalytic system based on osmium(IV) trihydride amidate derivatives, OsH_3_{κ^2^‐N,O‐[HNC(O)R]}(PiPr_3_)_2_. This system has demonstrated high efficiency as a catalyst, consistently delivering favorable outcomes in amide synthesis.

**SCHEME 23:**
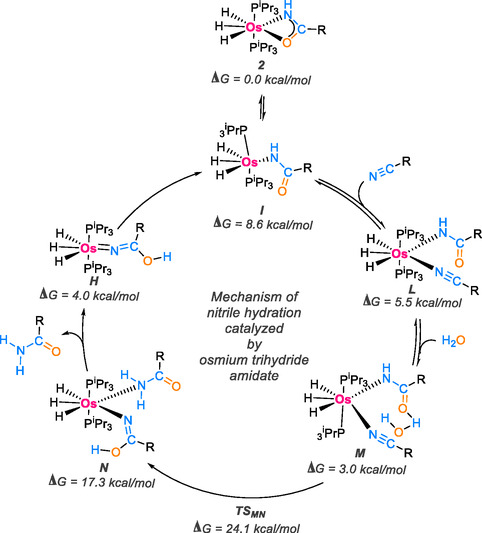
Proposed mechanism of nitrile hydration catalyzed by osmium trihydride amidate using DFT.

Through the application of DFT method, they presented a comprehensive reaction mechanism to distinguish experimental findings and elucidate the individual steps of the catalytic cycle effectively. This approach aims to enhance the understanding of both intermolecular and intramolecular interactions. The mechanism is illustrated in Scheme 23. The process initiates with the transformation of the coordination mode of the amidate ligand in compound **2** from κ2‐*N*,*O* to κ1‐N, creating a necessary vacancy that allows the entry of a nitrile molecule. This transformation results in the coordination of the nitrile to the κ1‐N‐amidate complex **I**, leading to the formation of the seven‐coordinate intermediate **L**, the key species in the catalysis. This intermediate undergoes an attack by an external water molecule. The free carbonyl group of the κ1‐N‐amidate ligand positions the water molecule close to the nitrile's C(sp) atom. Once positioned, the water molecule in the adduct **M** concertedly attacks the nitrile's C atom and the amidate's **N** atom. This concerted attack occurs through a six‐membered cyclic transition state, TSM‐N, involving interactions between the nitrile carbon, hydroxyl oxygen, and amidate nitrogen. This transition state is 24.1 kcal/mol higher in energy than the κ2‐amidate complex and leads to the formation of the κ1‐N‐iminolate complex **N**, which resembles complex **K** but contains a κ1‐N‐amide instead of a water ligand. The dissociation of the amide from complex **N** results in the hydroxoazavinylidene intermediate **H**, which subsequently tautomerizes to form the κ1‐N‐amidate complex I, thereby completing the catalytic cycle.

**SCHEME 24:**
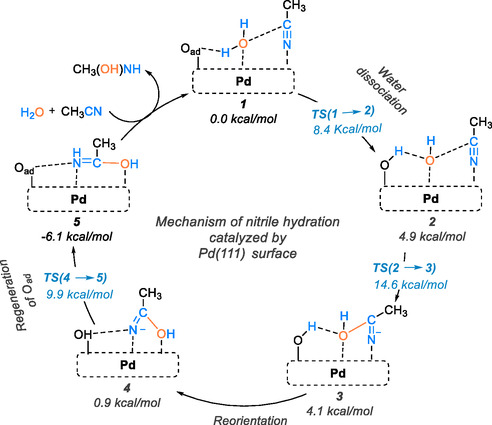
Proposed mechanism of nitrile hydration catalyzed by Pd(111) surface with adsorbed oxygen atoms.

On the other hand, Shimizu and colleagues proposed a reaction mechanism using the DFT method for the hydration of acetonitrile to acetamide on the surface of Pd/C [87], which is rich in adsorbed oxygen sites. The reaction mechanism and associated energetics are illustrated schematically in Scheme 24. The proposed mechanism begins with the dissociation of water. In the initial step (1 → 2), water dissociates on the surface, where O_ad_ acts as a Brønsted base, abstracting a proton from H_2_O and resulting in the formation of two OH groups on the Pd surface. In the most favorable coadsorption state of **1**, the water molecule occupies the top site, while the surface oxygen atom is located at the 3‐fold site. A strong hydrogen bond, measuring 1.923 Å, is formed between the water molecule and O_ad_ in state **1**. This occurs because O_ad_ is negatively charged (−0.65) due to interactions with the three nearest Pd atoms, which have an average positive charge of +0.21. The acetonitrile molecule is coadsorbed onto the top of the Pd surface and forms a weak bond with the oxygen atom of the water molecule. Subsequently, the water site deprotonates the O_ad_ molecule, requiring an activation energy of 8.38 kcal/mol. This water dissociation process is endothermic, with an energy change of 4.89 kcal/mol. The distance between the nitrile carbon atom and the OH group, derived from the water molecule, decreases from 3.994 Å in state **1** to 3.700 Å in state **2**. This indicates that the negatively charged OH group is attracting the nitrile carbon atom of the coadsorbed acetonitrile molecule. The nucleophilic attack by the OH species proceeds through TS (2 → 3), forming a C—O bond. In this transition state, the acetonitrile molecule shifts to a nearby top site, while the OH group remains almost in its original position. The C···O distance in the transition state is calculated to be 1.902 Å. The activation energy for the C—O bond formation is measured at 14.57 kcal/mol from **1**. This calculated activation energy aligns well with the experimental value of 17.44 kcal/mol. The overall reaction releases energy (is exothermic). The reorientation of the intermediate (3 → 4) occurs easily due to the Coulombic interaction between the remaining OH group and the negatively charged nitrogen atom, making this step exothermic by 3.17 kcal/mol. In the final step (4 → 5), the nitrogen atom accepts a proton from the OH group, regenerating the O_ad_ site. This step releases 7.0 kcal/mol of energy and has an activation energy of 8.98 kcal/mol. Consequently, the O_ad_ site is efficiently regenerated on the Pd surface, a key mechanistic feature of the catalyst contributing to its high turnover number. The resulting hydrated nitrile species is released into the solvent and easily converted into acetamide with the help of bridging water molecule.

## 7 Conclusion

This review article thoroughly examines the critical role of transition metal catalysts in the hydration reaction of a wide range of aliphatic, aromatic, and heteroaromatic nitriles, leading to the formation of biologically active amides. Researchers across different areas of chemistry have extensively investigated the efficacy of metal catalyst systems, highlighting their robust performance in yielding high‐quality amide products through nitrile hydration. The review categorizes the catalysts into three main types, with homogeneous catalysts standing out for their impressive efficiency. These catalysts leverage ligands like heterocyclic carbene and phosphoric organic auxiliary to enhance their activity through a donor–acceptor electron system. This system facilitates nitrile group activation by the metal, promoting water as the nucleophile in the reaction. Furthermore, heterogeneous catalysts utilize diverse organic or inorganic supports including polystyrene, titanium, silica, carbon, and magnetic materials. These supports possess specific surface properties, porosity, and thermal stability, crucial for enhancing the metal activity in coordinating the substrate and facilitating nitrile group activation for hydroxyl attack by water. The goal of these supports is to improve catalyst recycling and enable multiple reuses. Additionally, certain catalysts employ metal oxides that interact with water, creating an oxygen‐rich surface conducive to hydrogen bond formation between the substrate and surface. This accelerates nitrile group activation by water's hydroxyl, ultimately leading to amide product formation. Some catalysts require base activation in catalytic quantities, while others utilize inert gas‐containing mediums to enhance nitrile group activation and water nucleophilic attack. These heterogeneous catalysts are efficient, selective, and recoverable for converting nitriles to amides under eco‐friendly and acid‐ and base‐free conditions, delivering excellent yields. Separation from the reaction mixture is straightforward, either by simple filtration or by using an external magnetic pellet. Moreover, the catalytic system can be reused for several cycles without loss of efficiency, underscoring its sustainability and practicality. The review also delves into a detailed mechanistic study of base‐catalyzed hydration reactions using choline hydroxide and metal‐catalyzed hydration. By employing DFT methods, the review enhances the understanding of catalytic mechanisms, emphasizing the pivotal role of these methods in understanding and rationalizing experimental observations. The metal‐catalyzed hydration of nitriles into the vital amide functional group is reviving advancements enabling industrial adoption. Further works are tremendously necessary for scaling‐up this synthetic method to industrial‐scale manufacturing.

## 8 Future Prospects

This review highlights the use of various metal‐based catalysts for amide synthesis, emphasizing the most efficient catalysts, mechanistic insights obtained through DFT studies, and the pharmacological effects of the resulting amide products. Moving forward, this work can guide researchers in designing more efficient catalytic systems, exploring greener and milder reaction conditions, and developing amide derivatives with enhanced biological activities. Such insights will benefit not only researchers but also the broader scientific community by providing a comprehensive understanding of the interplay between catalyst design, reaction mechanism, and product functionality.

## Conflicts of Interest

The authors declare no conflicts of interest.

## Data Availability

The data that support the findings of this study are available from the corresponding author upon reasonable request.
